# 4-[4-(Dimethyl­amino)benzyl­idene]-2,6-dimethyl­cyclo­hexa-2,5-dienone

**DOI:** 10.1107/S1600536809030748

**Published:** 2009-08-08

**Authors:** Nathalie Hampel, Dorothea Richter, Armin R. Ofial, Herbert Mayr, Peter Mayer

**Affiliations:** a Ludwig-Maximilians-Universität, Department, Butenandtstrasse 5–13, 81377 München, Germany

## Abstract

The title compound, C_17_H_19_NO, crystallized with two mol­ecules per asymmetric unit. C—H⋯O hydrogen bonds lead to infinite chains along [100]. According to graph-set theory, the descriptor *C*_1_^1^(13)*C*_1_^1^(13) can be assigned.

## Related literature

For a related structure, see: Kawai *et al.* (2004 ▶). For background to graph set analysis, see: Bernstein *et al.* (1995 ▶); Etter *et al.* (1990 ▶). For the preparation, see: Richter *et al.* (2009 ▶).
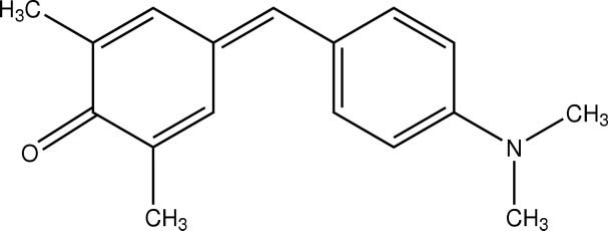


## Experimental

### 

#### Crystal data

C_17_H_19_NO
*M* *_r_* = 253.34
Monoclinic, 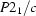

*a* = 14.5357 (3) Å
*b* = 7.2759 (2) Å
*c* = 27.5473 (5) Å
β = 104.6463 (14)°
*V* = 2818.74 (11) Å^3^
*Z* = 8
Mo *K*α radiation
μ = 0.07 mm^−1^
*T* = 200 K
0.24 × 0.20 × 0.19 mm

#### Data collection

Nonius KappaCCD diffractometer
Absorption correction: none
18635 measured reflections
5725 independent reflections
3685 reflections with *I* > 2σ(*I*)
*R*_int_ = 0.051

#### Refinement

*R*[*F* ^2^ > 2σ(*F* ^2^)] = 0.049
*wR*(*F* ^2^) = 0.131
*S* = 1.02
5725 reflections
351 parameters
H-atom parameters constrained
Δρ_max_ = 0.14 e Å^−3^
Δρ_min_ = −0.18 e Å^−3^

### 

Data collection: *COLLECT* (Hooft, 2004 ▶); cell refinement: *SCALEPACK* (Otwinowski & Minor, 1997 ▶); data reduction: *DENZO* (Otwinowski & Minor, 1997 ▶) and *SCALEPACK*; program(s) used to solve structure: *SIR97* (Altomare *et al.*, 1999 ▶); program(s) used to refine structure: *SHELXL97* (Sheldrick, 2008 ▶); molecular graphics: *ORTEP-3* (Farrugia, 1997 ▶) and *Mercury* (Macrae *et al.*, 2006 ▶); software used to prepare material for publication: *PLATON* (Spek, 2009 ▶).

## Supplementary Material

Crystal structure: contains datablocks I, global. DOI: 10.1107/S1600536809030748/fl2253sup1.cif

Structure factors: contains datablocks I. DOI: 10.1107/S1600536809030748/fl2253Isup2.hkl

Additional supplementary materials:  crystallographic information; 3D view; checkCIF report

## Figures and Tables

**Table 1:** Hydrogen-bond geometry (Å, °)

*D*—H⋯*A*	*D*—H	H⋯*A*	*D*⋯*A*	*D*—H⋯*A*
C17—H17*B*⋯O1^i^	0.98	2.51	3.456 (2)	163
C34—H34*B*⋯O2^i^	0.98	2.36	3.328 (2)	169

Symmetry code: (i) 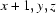.

**Fig. 1.**
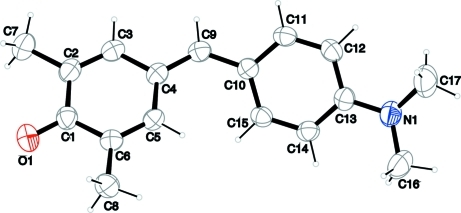
The molecular structure of the title compound (one molecule out of two of the asymmetric unit), with atom labels and anisotropic displacement ellipsoids (drawn at 50% probability level) for non-H atoms.

**Fig. 2.**
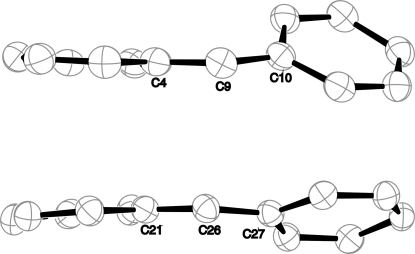
Schematic representation of the different orientations of the aromatic planes in the two molecules, viewed along the C–H bond of the linking group and parallel to the dienone ring planes. For clarity all atoms connected to the aromatic rings have been omitted.

**Fig. 3.**
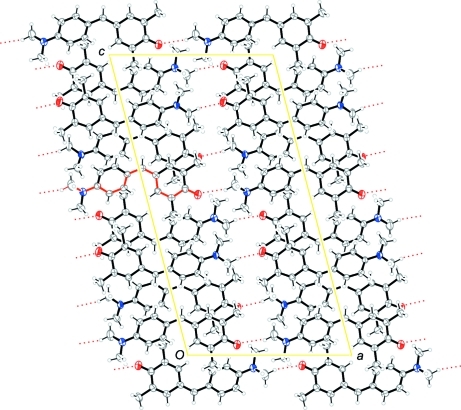
The packing of the title compound, viewed along [010]. Dashed lines indicate hydrogen bonds of the type C—H···O. The solid, red bonds link the 13 atoms involved in one of the two different hydrogen bonds leading to chains along [100].

## supplementary crystallographic information

### Comment

The asymmetric unit of (I) contains two complete molecules of the title compound. Figure 1 shows one of the two independent molecules.

A major difference between the two symmetrically independent molecules is found in the angle formed by the planes of the two C_6_-rings within a molecule. This angle is found to be 35.19 (7)° between the planes in one molecule but only 20.00 (7)° between the planes in the other molecule (Fig. 2). With a bulky naphthyl substituent at the C atom linking the two rings, an angle of 43.16 (6)° is observed [Kawai *et al.* (2004)].

The molecular packing, which is shown in Figure 3, is dominated by two C—H···O hydrogen bonds leading to infinite chains along [100]. Each of the chains is built up by 13 atoms and contains one donor atom and one acceptor atom. According to graph set theory [Bernstein *et al.* (1995), Etter *et al.* (1990)] the descriptor C_1_^1^(13)C_1_^1^(13) can be assigned. The strands are cross-linked by very weak C—H···C contacts with H···C distances of at least 2.79 Å (Table 1).

### Experimental

The title compound was prepared under an atmosphere of dry N_2_ from (4-hydroxy-3,5-dimethylphenyl)[4-(dimethylamino)phenyl]methanol [Richter *et al.* (2009)] (200 mg, 0.737 mmol) that was dissolved in dry CH_2_Cl_2_ (60 ml) and cooled to 0 °C. Then etheral HBF_4_-solution (0.110 ml, 0.811 mmol) was added at 0 °C. After 5 min, NEt_3_ (0.133 ml, 0.958 mmol) was added. The cooling bath was removed and stirring was continued for 3 h before the mixture was washed with water (3 times). The organic layer was dried (MgSO_4_) and the solvent was removed under reduced pressure. Crystals were obtained by slow cooling of a warm solution of the title compound in acetonitrile. Yield: 149 mg (80%), mp 127–128 °C.

### Refinement

The H atoms were positioned geometrically (C—H = 0.98 Å for CH_3_, 0.95 Å for CH) and treated as riding on their parent atoms [*U*_iso_(H) = 1.2*U*_eq_(C) for CH, *U*_iso_(H) = 1.5*U*_eq_(C) for CH_3_].

### Crystal data

**Table:**

C_17_H_19_NO	*F*(000) = 1088
*M**_r_* = 253.34	*D*_x_ = 1.194 (1) Mg m^−^^3^
Monoclinic, *P*2_1_/*c*	Mo *K*α radiation, λ = 0.71073 Å
Hall symbol: -P 2ybc	Cell parameters from 10514 reflections
*a* = 14.5357 (3) Å	θ = 3.1–26.4°
*b* = 7.2759 (2) Å	µ = 0.07 mm^−^^1^
*c* = 27.5473 (5) Å	*T* = 200 K
β = 104.6463 (14)°	Block, red
*V* = 2818.74 (11) Å^3^	0.24 × 0.20 × 0.19 mm
*Z* = 8	

### Data collection

**Table:**

Nonius KappaCCD diffractometer	3685 reflections with *I* > 2σ(*I*)
Radiation source: rotating anode	*R*_int_ = 0.051
MONTEL, graded multilayered X-ray optics	θ_max_ = 26.4°, θ_min_ = 3.2°
Detector resolution: 9 pixels mm^-1^	*h* = −18→18
CCD; rotation images; thick slices, φ/ω scan	*k* = −9→9
18635 measured reflections	*l* = −33→34
5725 independent reflections	

### Refinement

**Table:**

Refinement on *F*^2^	Primary atom site location: structure-invariant direct methods
Least-squares matrix: full	Secondary atom site location: difference Fourier map
*R*[*F*^2^ > 2σ(*F*^2^)] = 0.049	Hydrogen site location: inferred from neighbouring sites
*wR*(*F*^2^) = 0.131	H-atom parameters constrained
*S* = 1.02	*w* = 1/[σ^2^(*F*_o_^2^) + (0.058*P*)^2^ + 0.2713*P*] where *P* = (*F*_o_^2^ + 2*F*_c_^2^)/3
5725 reflections	(Δ/σ)_max_ = 0.001
351 parameters	Δρ_max_ = 0.14 e Å^−^^3^
0 restraints	Δρ_min_ = −0.18 e Å^−^^3^

### Special details

**Table:**

Geometry. All e.s.d.'s (except the e.s.d. in the dihedral angle between two l.s. planes) are estimated using the full covariance matrix. The cell e.s.d.'s are taken into account individually in the estimation of e.s.d.'s in distances, angles and torsion angles; correlations between e.s.d.'s in cell parameters are only used when they are defined by crystal symmetry. An approximate (isotropic) treatment of cell e.s.d.'s is used for estimating e.s.d.'s involving l.s. planes.
Refinement. Refinement of *F*^2^ against ALL reflections. The weighted *R*-factor *wR* and goodness of fit *S* are based on *F*^2^, conventional *R*-factors *R* are based on *F*, with *F* set to zero for negative *F*^2^. The threshold expression of *F*^2^ > 2*σ*(*F*^2^) is used only for calculating *R*-factors(gt) *etc*. and is not relevant to the choice of reflections for refinement. *R*-factors based on *F*^2^ are statistically about twice as large as those based on *F*, and *R*-factors based on all data will be even larger.

### Fractional atomic coordinates and isotropic or equivalent isotropic displacement parameters (Å^2^)

**Table:**

	*x*	*y*	*z*	*U*_iso_*/*U*_eq_	
O1	−0.31746 (8)	0.77657 (19)	0.46546 (5)	0.0626 (4)	
N1	0.37909 (9)	0.7322 (2)	0.45672 (5)	0.0497 (4)	
C1	−0.24976 (11)	0.7798 (2)	0.44563 (6)	0.0428 (4)	
C2	−0.26523 (11)	0.7680 (2)	0.39087 (6)	0.0441 (4)	
C3	−0.19006 (11)	0.7670 (2)	0.37098 (6)	0.0425 (4)	
H3	−0.2014	0.7639	0.3355	0.051*	
C4	−0.09286 (11)	0.7703 (2)	0.40056 (6)	0.0374 (4)	
C5	−0.07886 (11)	0.7914 (2)	0.45418 (5)	0.0372 (4)	
H5	−0.0157	0.7992	0.4747	0.045*	
C6	−0.15154 (11)	0.8003 (2)	0.47623 (5)	0.0372 (4)	
C7	−0.36678 (12)	0.7568 (3)	0.35963 (7)	0.0621 (5)	
H7A	−0.3675	0.7544	0.3240	0.093*	
H7B	−0.4022	0.8642	0.3664	0.093*	
H7C	−0.3964	0.6446	0.3682	0.093*	
C8	−0.13802 (12)	0.8369 (2)	0.53118 (6)	0.0477 (4)	
H8A	−0.0699	0.8458	0.5475	0.072*	
H8B	−0.1660	0.7362	0.5463	0.072*	
H8C	−0.1693	0.9527	0.5357	0.072*	
C9	−0.02032 (11)	0.7623 (2)	0.37695 (6)	0.0394 (4)	
H9	−0.0399	0.7699	0.3414	0.047*	
C10	0.08090 (10)	0.7442 (2)	0.39752 (5)	0.0350 (4)	
C11	0.14350 (11)	0.8184 (2)	0.37151 (5)	0.0395 (4)	
H11	0.1179	0.8716	0.3394	0.047*	
C12	0.24043 (11)	0.8172 (2)	0.39054 (6)	0.0413 (4)	
H12	0.2798	0.8735	0.3720	0.050*	
C13	0.28220 (11)	0.7344 (2)	0.43683 (6)	0.0385 (4)	
C14	0.22023 (11)	0.6473 (2)	0.46155 (5)	0.0395 (4)	
H14	0.2461	0.5826	0.4919	0.047*	
C15	0.12351 (11)	0.6539 (2)	0.44275 (5)	0.0370 (4)	
H15	0.0840	0.5956	0.4608	0.044*	
C16	0.41687 (13)	0.6822 (3)	0.50911 (7)	0.0713 (6)	
H16A	0.3946	0.7704	0.5305	0.107*	
H16B	0.4865	0.6838	0.5173	0.107*	
H16C	0.3949	0.5586	0.5149	0.107*	
C17	0.43927 (13)	0.8433 (3)	0.43384 (8)	0.0752 (6)	
H17A	0.4262	0.8142	0.3980	0.113*	
H17B	0.5061	0.8173	0.4500	0.113*	
H17C	0.4262	0.9737	0.4380	0.113*	
O2	−0.37910 (8)	0.25472 (19)	0.34392 (5)	0.0634 (4)	
N2	0.33071 (9)	0.24688 (19)	0.33231 (5)	0.0443 (4)	
C18	−0.30784 (12)	0.2279 (2)	0.32776 (6)	0.0444 (4)	
C19	−0.31707 (11)	0.1739 (2)	0.27539 (6)	0.0410 (4)	
C20	−0.23808 (10)	0.1486 (2)	0.25927 (6)	0.0386 (4)	
H20	−0.2453	0.1116	0.2254	0.046*	
C21	−0.14294 (10)	0.1744 (2)	0.29038 (5)	0.0348 (4)	
C22	−0.13547 (11)	0.2196 (2)	0.34218 (6)	0.0387 (4)	
H22	−0.0739	0.2329	0.3641	0.046*	
C23	−0.21173 (11)	0.2438 (2)	0.36091 (6)	0.0404 (4)	
C24	−0.41593 (11)	0.1501 (3)	0.24209 (7)	0.0547 (5)	
H24A	−0.4121	0.1181	0.2081	0.082*	
H24B	−0.4515	0.2651	0.2411	0.082*	
H24C	−0.4485	0.0516	0.2554	0.082*	
C25	−0.20350 (13)	0.2812 (3)	0.41543 (6)	0.0535 (5)	
H25A	−0.1363	0.2953	0.4331	0.080*	
H25B	−0.2309	0.1783	0.4300	0.080*	
H25C	−0.2379	0.3944	0.4188	0.080*	
C26	−0.06843 (10)	0.1560 (2)	0.26859 (5)	0.0348 (4)	
H26	−0.0867	0.1124	0.2350	0.042*	
C27	0.03223 (10)	0.1899 (2)	0.28665 (5)	0.0322 (4)	
C28	0.09255 (10)	0.1161 (2)	0.25908 (5)	0.0344 (4)	
H28	0.0648	0.0509	0.2291	0.041*	
C29	0.18928 (10)	0.1334 (2)	0.27339 (5)	0.0356 (4)	
H29	0.2268	0.0777	0.2538	0.043*	
C30	0.23412 (10)	0.2326 (2)	0.31684 (5)	0.0337 (4)	
C31	0.17424 (10)	0.3170 (2)	0.34342 (5)	0.0352 (4)	
H31	0.2015	0.3904	0.3720	0.042*	
C32	0.07772 (10)	0.2950 (2)	0.32885 (5)	0.0340 (4)	
H32	0.0399	0.3529	0.3479	0.041*	
C33	0.37588 (11)	0.3514 (3)	0.37678 (6)	0.0547 (5)	
H33A	0.3545	0.4795	0.3724	0.082*	
H33B	0.4451	0.3468	0.3820	0.082*	
H33C	0.3586	0.2987	0.4060	0.082*	
C34	0.39029 (11)	0.1576 (3)	0.30456 (7)	0.0613 (5)	
H34A	0.3789	0.0247	0.3038	0.092*	
H34B	0.4572	0.1823	0.3209	0.092*	
H34C	0.3751	0.2051	0.2702	0.092*	

### Atomic displacement parameters (Å^2^)

**Table:**

	*U*^11^	*U*^22^	*U*^33^	*U*^12^	*U*^13^	*U*^23^
O1	0.0423 (7)	0.0853 (11)	0.0637 (8)	−0.0013 (7)	0.0201 (6)	0.0060 (7)
N1	0.0368 (8)	0.0599 (10)	0.0510 (9)	−0.0005 (7)	0.0087 (7)	−0.0015 (7)
C1	0.0380 (10)	0.0389 (10)	0.0521 (10)	0.0030 (7)	0.0124 (8)	0.0058 (8)
C2	0.0385 (10)	0.0414 (11)	0.0488 (10)	0.0007 (8)	0.0045 (8)	0.0039 (8)
C3	0.0431 (10)	0.0449 (11)	0.0361 (9)	0.0003 (8)	0.0036 (7)	0.0007 (7)
C4	0.0378 (9)	0.0333 (9)	0.0386 (9)	0.0011 (7)	0.0053 (7)	−0.0010 (7)
C5	0.0364 (9)	0.0357 (10)	0.0375 (9)	0.0031 (7)	0.0055 (7)	−0.0011 (7)
C6	0.0435 (10)	0.0293 (9)	0.0387 (9)	0.0022 (7)	0.0099 (7)	0.0004 (7)
C7	0.0396 (10)	0.0792 (15)	0.0615 (12)	−0.0030 (10)	0.0018 (8)	0.0074 (10)
C8	0.0514 (11)	0.0480 (11)	0.0455 (10)	0.0000 (8)	0.0158 (8)	−0.0053 (8)
C9	0.0430 (10)	0.0396 (10)	0.0343 (8)	0.0027 (7)	0.0075 (7)	−0.0001 (7)
C10	0.0376 (9)	0.0342 (9)	0.0333 (8)	0.0021 (7)	0.0093 (7)	−0.0037 (7)
C11	0.0481 (11)	0.0388 (10)	0.0329 (8)	0.0061 (8)	0.0129 (7)	0.0021 (7)
C12	0.0440 (10)	0.0403 (10)	0.0444 (9)	0.0017 (8)	0.0199 (8)	0.0006 (8)
C13	0.0370 (9)	0.0378 (10)	0.0410 (9)	0.0029 (7)	0.0108 (7)	−0.0065 (7)
C14	0.0435 (10)	0.0419 (10)	0.0330 (8)	0.0071 (8)	0.0096 (7)	0.0011 (7)
C15	0.0418 (10)	0.0361 (10)	0.0356 (8)	0.0032 (7)	0.0144 (7)	0.0003 (7)
C16	0.0454 (11)	0.1020 (18)	0.0574 (12)	0.0055 (11)	−0.0036 (9)	−0.0013 (11)
C17	0.0449 (12)	0.0843 (17)	0.0953 (16)	−0.0098 (11)	0.0155 (11)	0.0064 (13)
O2	0.0438 (7)	0.0777 (10)	0.0766 (9)	−0.0019 (6)	0.0296 (7)	−0.0172 (7)
N2	0.0300 (8)	0.0539 (10)	0.0487 (8)	−0.0022 (6)	0.0097 (6)	−0.0082 (7)
C18	0.0397 (10)	0.0411 (11)	0.0572 (11)	−0.0015 (8)	0.0210 (8)	−0.0019 (8)
C19	0.0327 (9)	0.0379 (10)	0.0519 (10)	−0.0006 (7)	0.0099 (7)	−0.0001 (8)
C20	0.0365 (9)	0.0383 (10)	0.0398 (9)	−0.0006 (7)	0.0073 (7)	−0.0029 (7)
C21	0.0339 (9)	0.0324 (9)	0.0375 (8)	0.0014 (7)	0.0081 (7)	0.0002 (7)
C22	0.0337 (9)	0.0408 (10)	0.0400 (9)	−0.0033 (7)	0.0066 (7)	0.0003 (7)
C23	0.0436 (10)	0.0369 (10)	0.0437 (9)	−0.0036 (7)	0.0165 (8)	−0.0023 (7)
C24	0.0359 (10)	0.0649 (13)	0.0630 (11)	−0.0020 (9)	0.0117 (8)	−0.0051 (9)
C25	0.0613 (12)	0.0562 (13)	0.0482 (10)	−0.0044 (9)	0.0237 (9)	−0.0043 (9)
C26	0.0331 (9)	0.0354 (9)	0.0345 (8)	0.0013 (7)	0.0060 (7)	−0.0011 (7)
C27	0.0324 (9)	0.0325 (9)	0.0314 (8)	0.0024 (7)	0.0074 (6)	0.0025 (6)
C28	0.0370 (9)	0.0363 (9)	0.0296 (8)	−0.0012 (7)	0.0080 (6)	0.0004 (6)
C29	0.0365 (9)	0.0372 (10)	0.0366 (8)	0.0007 (7)	0.0159 (7)	−0.0013 (7)
C30	0.0284 (8)	0.0368 (10)	0.0360 (8)	0.0004 (7)	0.0084 (6)	0.0042 (7)
C31	0.0357 (9)	0.0363 (10)	0.0327 (8)	−0.0024 (7)	0.0070 (7)	−0.0022 (7)
C32	0.0335 (9)	0.0352 (9)	0.0342 (8)	0.0021 (7)	0.0104 (7)	−0.0008 (7)
C33	0.0362 (10)	0.0668 (14)	0.0552 (10)	−0.0054 (9)	0.0006 (8)	−0.0090 (9)
C34	0.0337 (10)	0.0803 (15)	0.0735 (13)	−0.0009 (9)	0.0201 (9)	−0.0154 (11)

### Geometric parameters (Å, °)

**Table:**

O1—C1	1.2406 (18)	O2—C18	1.2418 (18)
N1—C13	1.376 (2)	N2—C30	1.3642 (18)
N1—C17	1.447 (2)	N2—C34	1.446 (2)
N1—C16	1.455 (2)	N2—C33	1.450 (2)
C1—C2	1.470 (2)	C18—C23	1.468 (2)
C1—C6	1.471 (2)	C18—C19	1.468 (2)
C2—C3	1.341 (2)	C19—C20	1.344 (2)
C2—C7	1.511 (2)	C19—C24	1.507 (2)
C3—C4	1.442 (2)	C20—C21	1.443 (2)
C3—H3	0.9500	C20—H20	0.9500
C4—C9	1.373 (2)	C21—C26	1.371 (2)
C4—C5	1.447 (2)	C21—C22	1.441 (2)
C5—C6	1.347 (2)	C22—C23	1.347 (2)
C5—H5	0.9500	C22—H22	0.9500
C6—C8	1.500 (2)	C23—C25	1.501 (2)
C7—H7A	0.9800	C24—H24A	0.9800
C7—H7B	0.9800	C24—H24B	0.9800
C7—H7C	0.9800	C24—H24C	0.9800
C8—H8A	0.9800	C25—H25A	0.9800
C8—H8B	0.9800	C25—H25B	0.9800
C8—H8C	0.9800	C25—H25C	0.9800
C9—C10	1.443 (2)	C26—C27	1.4426 (19)
C9—H9	0.9500	C26—H26	0.9500
C10—C11	1.401 (2)	C27—C28	1.404 (2)
C10—C15	1.406 (2)	C27—C32	1.409 (2)
C11—C12	1.374 (2)	C28—C29	1.367 (2)
C11—H11	0.9500	C28—H28	0.9500
C12—C13	1.402 (2)	C29—C30	1.409 (2)
C12—H12	0.9500	C29—H29	0.9500
C13—C14	1.410 (2)	C30—C31	1.412 (2)
C14—C15	1.371 (2)	C31—C32	1.368 (2)
C14—H14	0.9500	C31—H31	0.9500
C15—H15	0.9500	C32—H32	0.9500
C16—H16A	0.9800	C33—H33A	0.9800
C16—H16B	0.9800	C33—H33B	0.9800
C16—H16C	0.9800	C33—H33C	0.9800
C17—H17A	0.9800	C34—H34A	0.9800
C17—H17B	0.9800	C34—H34B	0.9800
C17—H17C	0.9800	C34—H34C	0.9800
			
C13—N1—C17	119.38 (14)	C30—N2—C34	120.48 (13)
C13—N1—C16	119.30 (14)	C30—N2—C33	120.93 (13)
C17—N1—C16	116.99 (14)	C34—N2—C33	118.59 (13)
O1—C1—C2	121.20 (15)	O2—C18—C23	120.91 (15)
O1—C1—C6	120.85 (15)	O2—C18—C19	121.12 (15)
C2—C1—C6	117.93 (14)	C23—C18—C19	117.94 (13)
C3—C2—C1	119.39 (14)	C20—C19—C18	119.19 (14)
C3—C2—C7	123.14 (16)	C20—C19—C24	123.09 (15)
C1—C2—C7	117.47 (15)	C18—C19—C24	117.72 (14)
C2—C3—C4	123.54 (15)	C19—C20—C21	123.81 (14)
C2—C3—H3	118.2	C19—C20—H20	118.1
C4—C3—H3	118.2	C21—C20—H20	118.1
C9—C4—C3	119.46 (14)	C26—C21—C22	125.83 (13)
C9—C4—C5	124.14 (14)	C26—C21—C20	118.16 (13)
C3—C4—C5	116.32 (14)	C22—C21—C20	116.00 (13)
C6—C5—C4	122.78 (14)	C23—C22—C21	123.07 (14)
C6—C5—H5	118.6	C23—C22—H22	118.5
C4—C5—H5	118.6	C21—C22—H22	118.5
C5—C6—C1	119.69 (14)	C22—C23—C18	119.83 (14)
C5—C6—C8	123.10 (14)	C22—C23—C25	122.80 (15)
C1—C6—C8	117.18 (14)	C18—C23—C25	117.34 (14)
C2—C7—H7A	109.5	C19—C24—H24A	109.5
C2—C7—H7B	109.5	C19—C24—H24B	109.5
H7A—C7—H7B	109.5	H24A—C24—H24B	109.5
C2—C7—H7C	109.5	C19—C24—H24C	109.5
H7A—C7—H7C	109.5	H24A—C24—H24C	109.5
H7B—C7—H7C	109.5	H24B—C24—H24C	109.5
C6—C8—H8A	109.5	C23—C25—H25A	109.5
C6—C8—H8B	109.5	C23—C25—H25B	109.5
H8A—C8—H8B	109.5	H25A—C25—H25B	109.5
C6—C8—H8C	109.5	C23—C25—H25C	109.5
H8A—C8—H8C	109.5	H25A—C25—H25C	109.5
H8B—C8—H8C	109.5	H25B—C25—H25C	109.5
C4—C9—C10	130.31 (14)	C21—C26—C27	132.61 (14)
C4—C9—H9	114.8	C21—C26—H26	113.7
C10—C9—H9	114.8	C27—C26—H26	113.7
C11—C10—C15	115.83 (13)	C28—C27—C32	115.29 (13)
C11—C10—C9	119.57 (13)	C28—C27—C26	117.75 (13)
C15—C10—C9	124.60 (14)	C32—C27—C26	126.93 (13)
C12—C11—C10	122.60 (14)	C29—C28—C27	123.11 (13)
C12—C11—H11	118.7	C29—C28—H28	118.4
C10—C11—H11	118.7	C27—C28—H28	118.4
C11—C12—C13	121.08 (14)	C28—C29—C30	120.92 (13)
C11—C12—H12	119.5	C28—C29—H29	119.5
C13—C12—H12	119.5	C30—C29—H29	119.5
N1—C13—C12	122.09 (14)	N2—C30—C29	121.54 (13)
N1—C13—C14	121.14 (14)	N2—C30—C31	121.71 (13)
C12—C13—C14	116.74 (14)	C29—C30—C31	116.75 (13)
C15—C14—C13	121.43 (14)	C32—C31—C30	121.21 (13)
C15—C14—H14	119.3	C32—C31—H31	119.4
C13—C14—H14	119.3	C30—C31—H31	119.4
C14—C15—C10	122.08 (14)	C31—C32—C27	122.56 (13)
C14—C15—H15	119.0	C31—C32—H32	118.7
C10—C15—H15	119.0	C27—C32—H32	118.7
N1—C16—H16A	109.5	N2—C33—H33A	109.5
N1—C16—H16B	109.5	N2—C33—H33B	109.5
H16A—C16—H16B	109.5	H33A—C33—H33B	109.5
N1—C16—H16C	109.5	N2—C33—H33C	109.5
H16A—C16—H16C	109.5	H33A—C33—H33C	109.5
H16B—C16—H16C	109.5	H33B—C33—H33C	109.5
N1—C17—H17A	109.5	N2—C34—H34A	109.5
N1—C17—H17B	109.5	N2—C34—H34B	109.5
H17A—C17—H17B	109.5	H34A—C34—H34B	109.5
N1—C17—H17C	109.5	N2—C34—H34C	109.5
H17A—C17—H17C	109.5	H34A—C34—H34C	109.5
H17B—C17—H17C	109.5	H34B—C34—H34C	109.5
			
O1—C1—C2—C3	−178.22 (16)	O2—C18—C19—C20	179.15 (16)
C6—C1—C2—C3	3.3 (2)	C23—C18—C19—C20	−2.7 (2)
O1—C1—C2—C7	1.5 (2)	O2—C18—C19—C24	−0.6 (2)
C6—C1—C2—C7	−176.94 (15)	C23—C18—C19—C24	177.49 (15)
C1—C2—C3—C4	2.3 (3)	C18—C19—C20—C21	−1.1 (2)
C7—C2—C3—C4	−177.45 (16)	C24—C19—C20—C21	178.72 (15)
C2—C3—C4—C9	177.69 (16)	C19—C20—C21—C26	−175.74 (15)
C2—C3—C4—C5	−5.3 (2)	C19—C20—C21—C22	3.6 (2)
C9—C4—C5—C6	179.44 (15)	C26—C21—C22—C23	176.92 (16)
C3—C4—C5—C6	2.6 (2)	C20—C21—C22—C23	−2.4 (2)
C4—C5—C6—C1	2.9 (2)	C21—C22—C23—C18	−1.3 (2)
C4—C5—C6—C8	−175.02 (15)	C21—C22—C23—C25	176.74 (16)
O1—C1—C6—C5	175.68 (15)	O2—C18—C23—C22	−178.00 (16)
C2—C1—C6—C5	−5.8 (2)	C19—C18—C23—C22	3.9 (2)
O1—C1—C6—C8	−6.3 (2)	O2—C18—C23—C25	3.9 (2)
C2—C1—C6—C8	172.17 (15)	C19—C18—C23—C25	−174.24 (15)
C3—C4—C9—C10	−172.71 (15)	C22—C21—C26—C27	−6.6 (3)
C5—C4—C9—C10	10.5 (3)	C20—C21—C26—C27	172.68 (15)
C4—C9—C10—C11	−151.45 (17)	C21—C26—C27—C28	165.06 (16)
C4—C9—C10—C15	29.2 (3)	C21—C26—C27—C32	−16.9 (3)
C15—C10—C11—C12	−5.2 (2)	C32—C27—C28—C29	4.1 (2)
C9—C10—C11—C12	175.42 (14)	C26—C27—C28—C29	−177.62 (13)
C10—C11—C12—C13	2.4 (2)	C27—C28—C29—C30	−1.7 (2)
C17—N1—C13—C12	10.3 (2)	C34—N2—C30—C29	−0.8 (2)
C16—N1—C13—C12	166.30 (17)	C33—N2—C30—C29	179.24 (15)
C17—N1—C13—C14	−171.83 (16)	C34—N2—C30—C31	179.45 (15)
C16—N1—C13—C14	−15.9 (2)	C33—N2—C30—C31	−0.5 (2)
C11—C12—C13—N1	−179.73 (15)	C28—C29—C30—N2	178.22 (14)
C11—C12—C13—C14	2.3 (2)	C28—C29—C30—C31	−2.0 (2)
N1—C13—C14—C15	177.92 (14)	N2—C30—C31—C32	−177.05 (14)
C12—C13—C14—C15	−4.1 (2)	C29—C30—C31—C32	3.2 (2)
C13—C14—C15—C10	1.3 (2)	C30—C31—C32—C27	−0.7 (2)
C11—C10—C15—C14	3.3 (2)	C28—C27—C32—C31	−2.9 (2)
C9—C10—C15—C14	−177.29 (14)	C26—C27—C32—C31	179.02 (14)

### Hydrogen-bond geometry (Å, °)

**Table:**

*D*—H···*A*	*D*—H	H···*A*	*D*···*A*	*D*—H···*A*
C17—H17B···O1^i^	0.98	2.51	3.456 (2)	163
C34—H34B···O2^i^	0.98	2.36	3.328 (2)	169

Symmetry codes: (i) *x*+1, *y*, *z*.
